# A longitudinal plasma metabolomics dataset of sepsis-associated liver injury across multiple clinical time points

**DOI:** 10.1038/s41597-026-08148-5

**Published:** 2026-08-24

**Authors:** Huan Liu, Jing Yang, Dong Leng, Yihui Wang, Enqiang Mao, Erzhen Chen, Ying Chen, Zhitao Yang

**Affiliations:** 1 Department of Emergency, Ruijin Hospital, Shanghai Jiao Tong University School of Medicine, Shanghai, 200025 China; 2 Shanghai Institute of Aviation Medicine, Ruijin Hospital, Shanghai Jiao Tong University School of Medicine, Shanghai, 200025 China; 3 Emergency & Critical Care Medicine Center, Ruijin Hospital, Shanghai Jiao Tong University School of Medicine, Shanghai, 200025 China; 4 College of Animal Science and Technology, Nanjing Agricultural University, Nanjing, 210095 China

## Abstract

Sepsis-associated liver injury is a severe complication of sepsis characterized by substantial metabolic alterations, yet longitudinal metabolomics resources for this condition remain limited. Here, we present an LC-MS-based longitudinal metabolomics dataset comprising 88 blood samples collected from healthy controls and sepsis patients with or without liver injury across multiple ICU-associated clinical time points. Comprehensive metabolomic profiling generated 18,247 high-quality metabolic features and 2,894 annotated metabolites, providing a resource for investigating temporal metabolite patterns with annotations related to nucleotide, lipid, and bile acid metabolism across clinical groups and sampling time points. Exploratory analytical approaches, including temporal clustering, weighted correlation network analysis, and regularized regression-based feature selection, were applied to facilitate characterization of longitudinal metabolic patterns within the dataset. This dataset provides a reusable resource for future studies on longitudinal metabolic dynamics, biomarker validation, integrative multi-omics analyses, and methodological development in sepsis and critical care research.

## Background & Summary

Sepsis is a life-threatening systemic condition caused by a dysregulated host response to infection, leading to profound organ dysfunction and high mortality rates^1,2^. As one of the most critical challenges in modern intensive care medicine, sepsis affects approximately 31.5 million patients worldwide each year, with mortality rates ranging from 30% to 50% in general cases and escalating to 60–80% in septic shock patients^3,4^. While the pathophysiology of sepsis was initially attributed to uncontrolled systemic inflammation and infection, research in recent years has suggested a complex interplay of immunological, coagulative, and metabolic disturbances that collectively drive disease progression^5–8^.

The liver is highly susceptible to dysfunction during sepsis because of its central roles in systemic metabolism, detoxification, and immune regulation^9^. Sepsis-associated liver injury (SLI) has been reported in approximately 34–46% of sepsis cases and is associated with increased mortality^9–11^. Previous studies have reported changes in inflammatory responses, mitochondrial function, bile acid metabolism, and metabolic profiles in SLI^10–17^. However, the longitudinal metabolic characteristics associated with sepsis progression and liver injury remain incompletely characterized.

Metabolomics has emerged as a useful approach for characterizing systemic metabolic alterations during sepsis and organ dysfunction. Previous studies have reported perturbations in bile acid metabolism, lipid metabolism, amino acid metabolism, and immune-associated metabolic pathways in sepsis patients^18–24^. However, most available metabolomics studies are cross-sectional and lack longitudinal sampling across disease progression stages. Publicly available longitudinal metabolomics datasets for sepsis-associated liver injury remain limited, restricting opportunities for temporal trajectory analysis, biomarker validation, and integrative multi-omics investigations.

**Fig. 1:**
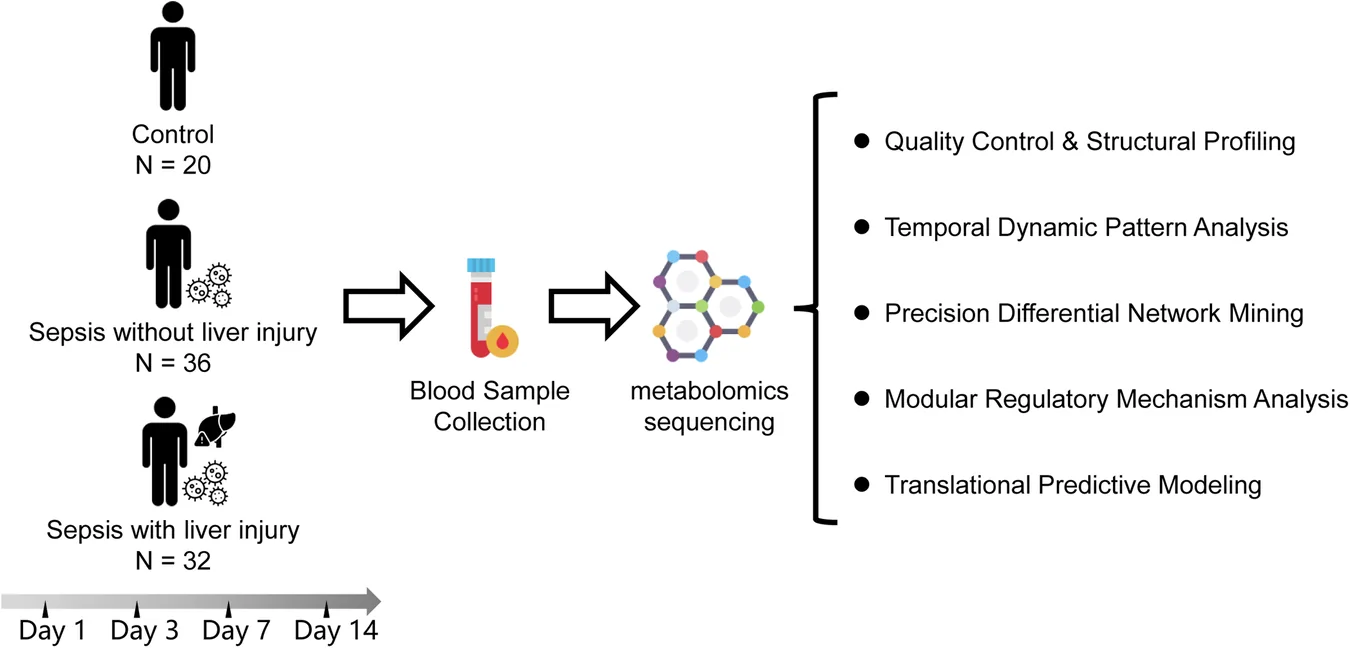
Experimental design. Human blood samples were collected from controls and sepsis patients (with/without liver injury) at four time points (D1, D3, D7, and D14). Untargeted metabolomics profiling was conducted using LC-MS, followed by multi-faceted data analysis.

This study provides a longitudinal LC-MS metabolomics dataset for investigating metabolic alterations associated with sepsis progression and sepsis-associated liver injury (Fig. 1). The dataset includes repeated longitudinal sampling, comprehensive sample metadata, raw and processed LC-MS files, metabolite annotations, and accompanying analytical results (Tables 1–6). These resources may facilitate future studies on temporal metabolic dynamics, biomarker validation, systems biology, and integrative multi-omics analyses in critical care research.

**Table 1:** Basic information of the 37 participants.

Basic information	Control	Sepsis with liver injury	Sepsis without liver injury
Number	20	8	9
Age (years)	65.65 ± 3.23	59.75 ± 14.37	70.33 ± 13.33
Gender (male/female)	10/10	4/4	6/3
BMI (kg/m2)	21.37 ± 1.83	22.65 ± 5.67	22.64 ± 4.91
LOS (days)	NA	32.50 ± 11.68	30.89 ± 12.39

BMI: body mass index; LOS: Length of Stay; NA: not applicable.

**Table 2:** Primary infection site information of 17 patients.

Primary infection site	Sepsis with liver injury	Sepsis without liver injury
Pancreas	3	3
Biliary tract	0	1
Gastrointestinal tract	1	1
Abdominal cavity	0	2
Liver	0	1
Lung	4	0
Urinary tract	1	0

**Table 3:** Comorbidities information of 17 patients.

Comorbidities	Sepsis with liver injury	Sepsis without liver injury
Hypertension	5	3
Diabetes mellitus	3	2
Coronary artery disease	2	0
Hyperlipidemia	1	2
Fatty liver disease	0	0
Stroke	1	0
Cardiac arrhythmia	1	1
Renal disease	1	1
Respiratory disease	1	1

**Table 4:** Hematological information of 17 patients.

Hematological	Sepsis with liver injury	Sepsis without liver injury
White blood cell count (×10⁹/L)	14.98 ± 6.73	13.93 ± 6.10
Red blood cell count (×10¹²/L)	3.34 ± 0.71	3.52 ± 0.45
Hemoglobin (g/L)	104.75 ± 23.00	106.11 ± 13.58
Platelet count (×10⁹/L)	122.87 ± 55.39	170.44 ± 90.01

**Table 5:** Liver function information of 17 patients.

Liver function	Sepsis with liver injury	Sepsis without liver injury
ALT (U/L)	297.38 ± 647.32	55.00 ± 50.39
AST (U/L)	299.25 ± 545.58	57.78 ± 45.85
ALP (U/L)	81.50 ± 29.29	110.00 ± 76.59
GGT (U/L)	71.38 ± 60.02	78.67 ± 74.36
Total bilirubin (μmol/L)	64.25 ± 20.80	13.80 ± 7.13
Direct bilirubin (μmol/L)	33.11 ± 14.21	4.33 ± 2.50

ALT: Alanine aminotransferase; AST: Aspartate aminotransferase; ALP: Alkaline phosphatase; GGT: Gamma-glutamyl transferase.

**Table 6:** Renal function and electrolytes information of 17 patients.

Renal function and electrolytes	Sepsis with liver injury	Sepsis without liver injury
Creatinin-e (μmol/L)	129.75 ± 97.56	155.44 ± 203.10
Urea (mmol/L)	10.66 ± 5.22	27.04 ± 39.79
Potassium (K⁺) (mmol/L)	4.00 ± 0.62	3.89 ± 0.53
Sodium (Na⁺) (mmol/L)	145.13 ± 7.32	142.78 ± 5.47
Chloride (Cl⁻) (mmol/L)	108.88 ± 6.11	106.25 ± 5.55
Calcium (Ca²⁺) (mmol/L)	1.94 ± 0.35	1.79 ± 0.55
Phosphate (mmol/L)	0.74 ± 0.20	1.23 ± 1.04
CRP (mg/L)	213.38 ± 87.76	166.89 ± 103.39
PT (s)	18.84 ± 6.60	14.80 ± 1.63
APTT (s)	39.60 ± 15.75	33.71 ± 7.17
Fibrinogen (g/L)	4.05 ± 2.04	4.15 ± 1.92
D-Dimer (mg/L)	5.65 ± 3.80	8.21 ± 7.13
FDP (mg/L)	16.90 ± 10.65	22.64 ± 13.95

CRP: C-reactive protein; PT: Prothrombin time; APTT: Activated partial thromboplastin time; FDP: Fibrin degradation products.

**Fig. 2:**
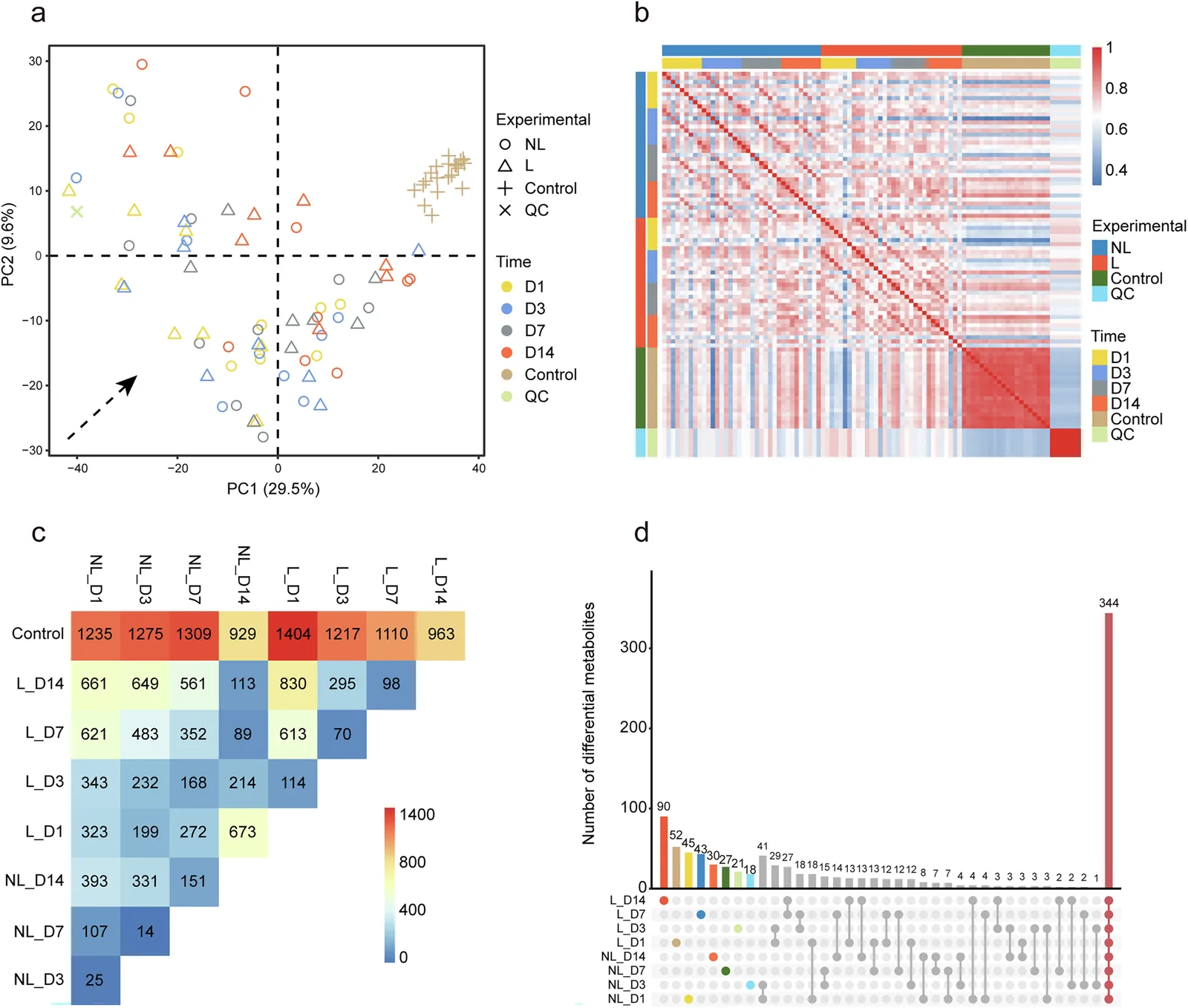
Comprehensive overview of metabolomics sequencing data. (**a**) Principal component analysis (PCA) clustering analysis based on metabolite abundance values. NL: sepsis without liver injury group; L: sepsis with liver injury group; Control: control group; QC: quality control group; D1: day 1; D3: day 3; D7: day 7; D14: day 14. (**b**) Spearman’s heatmap illustrating correlation between metabolomics sequencing samples. (**c**) Heatmap of the number of differential metabolites in pairwise comparisons across nine groups. Threshold criteria: OPLS-DA VIP > 1 and *P* < 0.05. (**d**) Upset plot of differential metabolites between NL/L groups and the control group across four time points. The leftmost colored bars represent the number of unique differential metabolites for each group. The rightmost bar indicates the intersection of differential metabolites shared across all eight groups compared to the control. The remaining gray bars represent unique intersections of differential metabolites between any two groups and the control.

Our metabolomic analysis identified 18,247 high-quality peaks with distinct chromatographic profiles and uniform distribution. Database matching revealed 2,894 metabolites for subsequent investigations. Principal component analysis (PCA) suggested temporal separation trends of samples from D1 to D14 along the diagonal axis, with controls and quality control samples forming distinct clusters (Fig. 2a). Inter-sample correlation analysis indicated reproducible results among biological replicates, with higher correlations among control groups (Spearman’s correlation coefficient > 0.87) (Fig. 2b). Pairwise orthogonal partial least squares-discriminant analysis (OPLS-DA) indicated relatively limited metabolic separation between adjacent time points within the same group (Fig. 2c). Notably, 344 commonly differential metabolites were observed across sepsis-related comparisons when compared to controls (Fig. 2d).

**Fig. 3:**
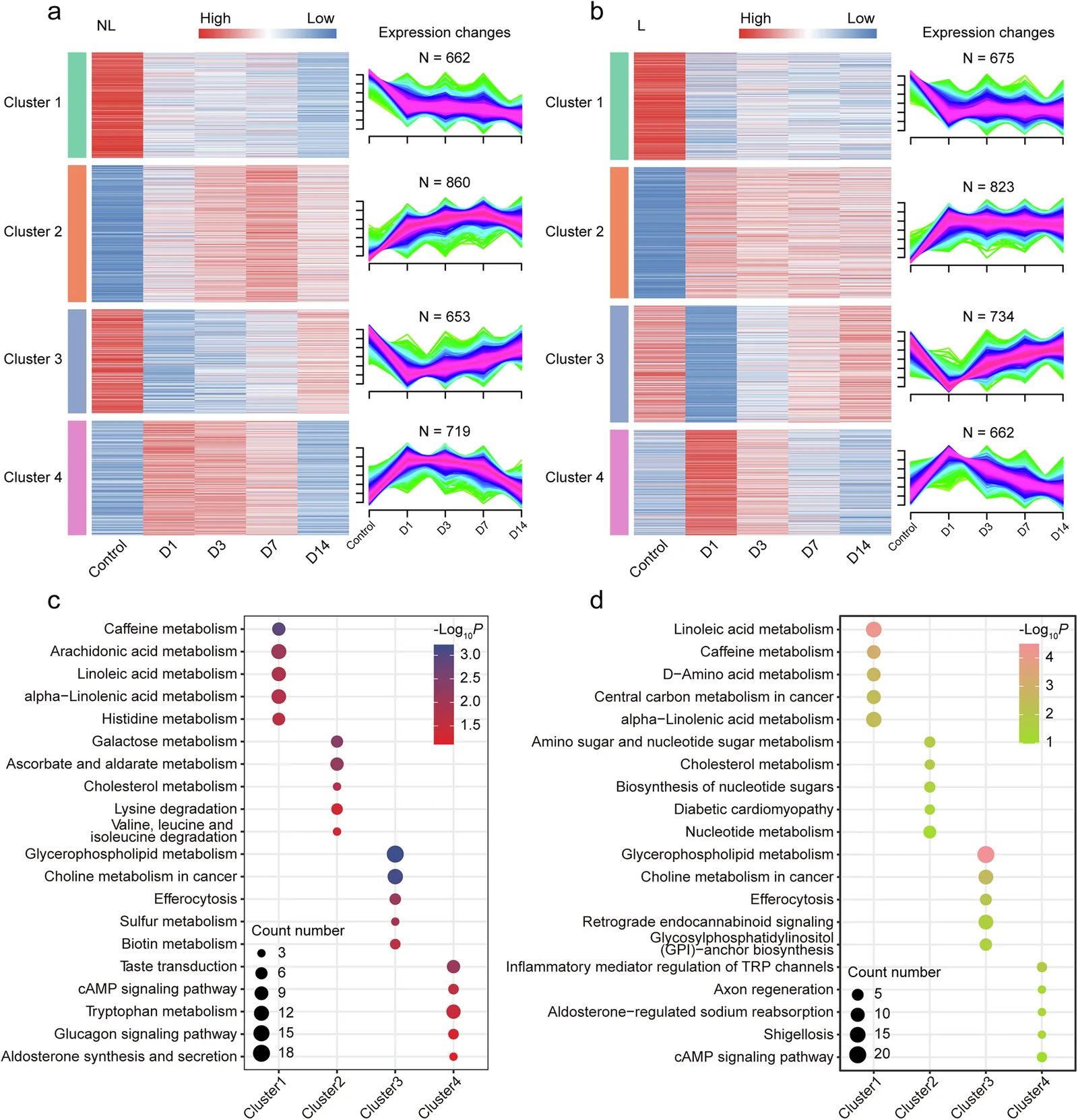
Dynamic metabolic abundance landscapes in NL and L Groups. (**a-b**) Heatmaps and line charts displaying fuzzy c-means clustering results of four distinct temporal patterns (Clusters 1–4) in metabolite abundance for the NL group (**a**) and L group (**b**). Purple and red lines represent metabolites with high membership values. The x-axis indicates the control group and four sampling time points, while the y-axis shows normalized abundance values from Mfuzz analysis. (**c-d**) Bubble plots illustrating KEGG enrichment analysis of metabolites in different clusters for the NL group (**c**) and L group (**d**). Color gradient denotes *P* values, and circle size corresponds to the number of metabolites.

Exploratory fuzzy c-means clustering analysis grouped metabolites into four major temporal abundance patterns across longitudinal sampling points in both NL and L groups (Fig. 3a, b). Cluster 1 metabolites exhibited a decreasing temporal abundance pattern across the sampled time points. Pathway enrichment analysis identified over-represented pathways including “caffeine metabolism” in the NL group and “linoleic acid metabolism” in the L group. Cluster 2 metabolites exhibited an increasing temporal abundance pattern across the sampled time points. Pathway annotation indicated enrichment for pathways including “galactose metabolism” in the NL group and “amino sugar and nucleotide sugar metabolism” in the L group. Cluster 3 metabolites showed higher relative abundance in controls, followed by decreased abundance at D1 and increased abundance at later sampled time points. Pathway annotation indicated representation of “glycerophospholipid metabolism” among the annotated pathways in both groups. Cluster 4 metabolites showed lower relative abundance in controls, followed by increased abundance at D1 and decreased abundance at subsequent sampled time points. Pathway enrichment analysis identified “taste transduction” in the NL group and “inflammatory mediator regulation of TRP channels” in the L group (Fig. 3c, d).

**Fig. 4:**
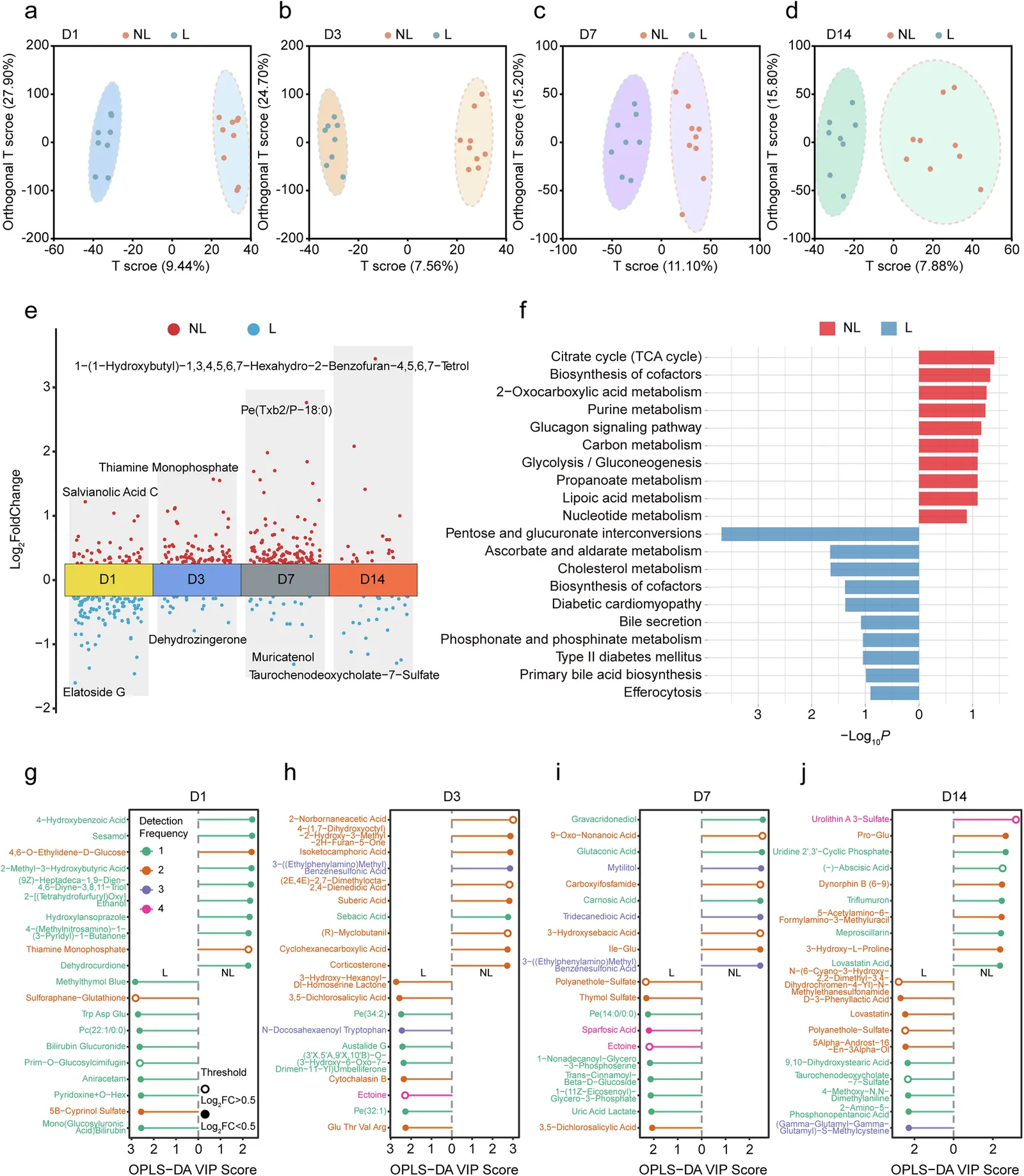
Differential metabolite analysis in NL and L groups across four time points. (**a-d**) Orthogonal partial least squares discriminant analysis (OPLS-DA) supervised clustering analysis of metabolomics data for NL and L groups at D1 (**a**), D3 (**b**), D7 (**c**), and D14 (**d**) based on metabolite abundance values. (**e**) Multiple volcano plots displaying differential metabolites in NL and L groups at each time point. The most significantly altered metabolite (highest log_2_ fold change) per group is labeled. Threshold: OPLS-DA VIP > 1 and *P* < 0.05. (**f**) Bidirectional bar plot of KEGG pathway enrichment for all significant differential metabolites across four time points. The x-axis denotes *P* values. (**g-j**) Bidirectional lollipop charts showing the top 10 OPLS-DA VIP metabolites for NL and L groups at D1 (**g**), D3 (**h**), D7 (**i**), and D14 (**j**). The x-axis denotes OPLS-DA VIP score; color gradient corresponds frequency of detection as a significant differential metabolite across time points; filled/hollow circles show log_2_ fold change > 0.5 (hollow) or < 0.5 (filled).

To characterize metabolite differences observed between patients with and without liver injury, we performed pairwise OPLS-DA comparisons between the NL and L groups at each time point (Fig. 4a-d). The metabolite exhibiting the largest fold change was 1-(1-Hydroxybutyl)-1,3,4,5,6,7-Hexahydro-2-Benzofuran-4,5,6,7-Tetrol at D14 in the NL group, and Elatoside G at D1 in the L group (Fig. 4e). Pathway annotation of differential metabolites indicated enrichment for the citrate cycle pathway in the NL group and pentose and glucuronate interconversion pathways in the L group (Fig. 4f). Notably, Ectoine was consistently upregulated across all time points in the L group, with a log₂FC > 0.5 at D3 and D7, and ranked among the top 10 variable importance index (VIP) scores. In contrast, Urolithin A 3-Sulfate was significantly upregulated in the NL group at all time points, achieving the highest VIP score at D14 with a log₂FC > 0.5 (Fig. 4g-j).

**Fig. 5:**
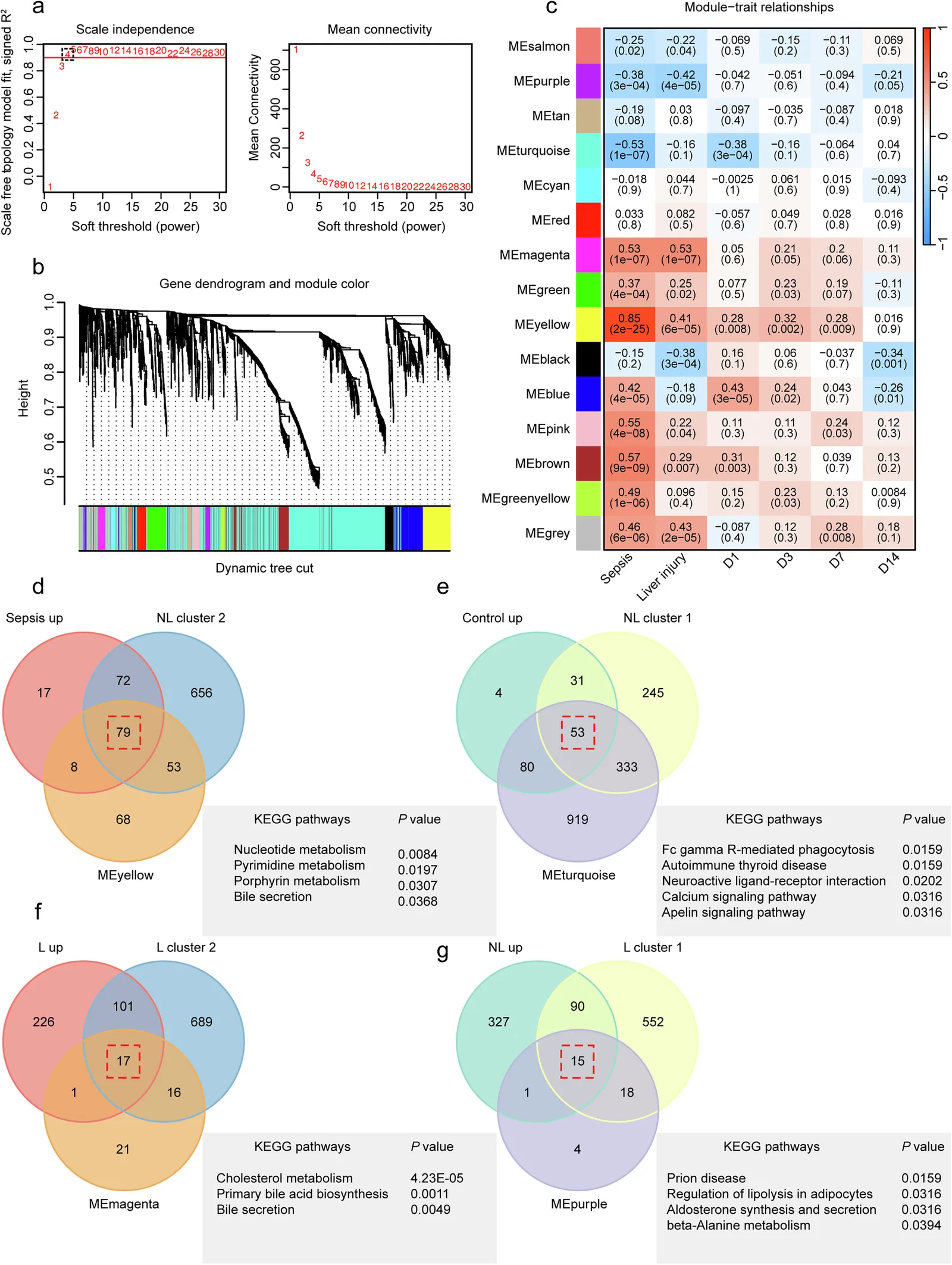
Weighted Gene Co-Expression Network Analysis (WGCNA) of all samples. (**a**) Network topology analysis determined the optimal soft-thresholding power (β) by evaluating scale independence and mean connectivity. The selected power value achieved a scale-free topology fit with R² > 0.9 while maintaining reasonable network connectivity. (**b**) Hierarchical clustering of metabolites based on topological overlap dissimilarity generated a dendrogram that revealed distinct co-expression patterns. Dynamic branch cutting identified several color-coded modules, with each module representing a group of highly interconnected metabolites. The grey module contained metabolites that did not belong to any specific co-expression group. (**c**) Heatmaps of the correlation between module representative metabolite expression patterns and trait data. The color gradient from red to blue represented the strength and direction of these associations, with detailed numerical values showing both correlation coefficients and corresponding *P* values for each module-trait pair. (**d-e**) Venn diagram shows the intersection of the three groups of metabolites that are positively (**d**) and negatively (**e**) correlat**e**d with sepsis respectively. The grey panel in the lower right presents the KEGG enrichment analysis results of the three groups of intersecting metabolites, including their pathways and *P* values. (**f-g**) Venn diagram shows the intersection of the three groups of metabolites that are positively (**f**) and negatively (**g**) correlated with liver injury respectively. The grey panel in the lower right presents the KEGG enrichment analysis results of the three groups of intersecting metabolites, including their pathways and *P* values.

Using weighted gene co-expression network analysis (WGCNA), we identified metabolic modules showing different correlation patterns across clinical groups and liver injury status. The optimal soft threshold was determined to be 4 (Fig. 5a). Cluster analysis revealed 15 distinct modules, including 14 functional modules and 1 non-functional grey module (Fig. 5b). Module-trait correlation analysis demonstrated correlation patterns: the yellow module showed potential relationships with sepsis; the magenta module exhibited potential relationships with liver injury; the turquoise module displayed potential relationships with sepsis that progressively strengthened from D1 to D14; the purple module showed potential relationships with liver injury; while the blue module demonstrated progressively decreasing correlation from D1 to D14 (Fig. 5c).

To identify exploratory metabolic signatures associated with sepsis and sepsis-associated liver injury, we performed integrative analysis of metabolites derived from multiple analytical approaches. The sepsis-positive correlation signature comprised 79 consensus metabolites selected through three exploratory criteria: metabolites consistently upregulated in sepsis groups compared to controls, metabolites showing progressive increases from controls to D14 in NL group cluster 2, and metabolites belonging to the sepsis-associated yellow module, with pathway annotation indicating enrichment for “nucleotide metabolism” (Fig. 5d). Conversely, the exploratory sepsis-negative correlation signature included 53 overlapping metabolites selected from multiple analytical outputs, with pathway annotation indicating enrichment for “Fc gamma R-mediated phagocytosis” (Fig. 5e). For liver injury-associated exploratory signatures, 17 metabolites were selected through intersection analysis of multiple analytical outputs, with pathway annotation indicating enrichment for “cholesterol metabolism” (Fig. 5f). Additionally, 15 metabolites from the liver injury-associated exploratory signature were selected through intersection analysis, with pathway annotation indicating enrichment for “prion disease” pathways (Fig. 5g).

**Fig. 6:**
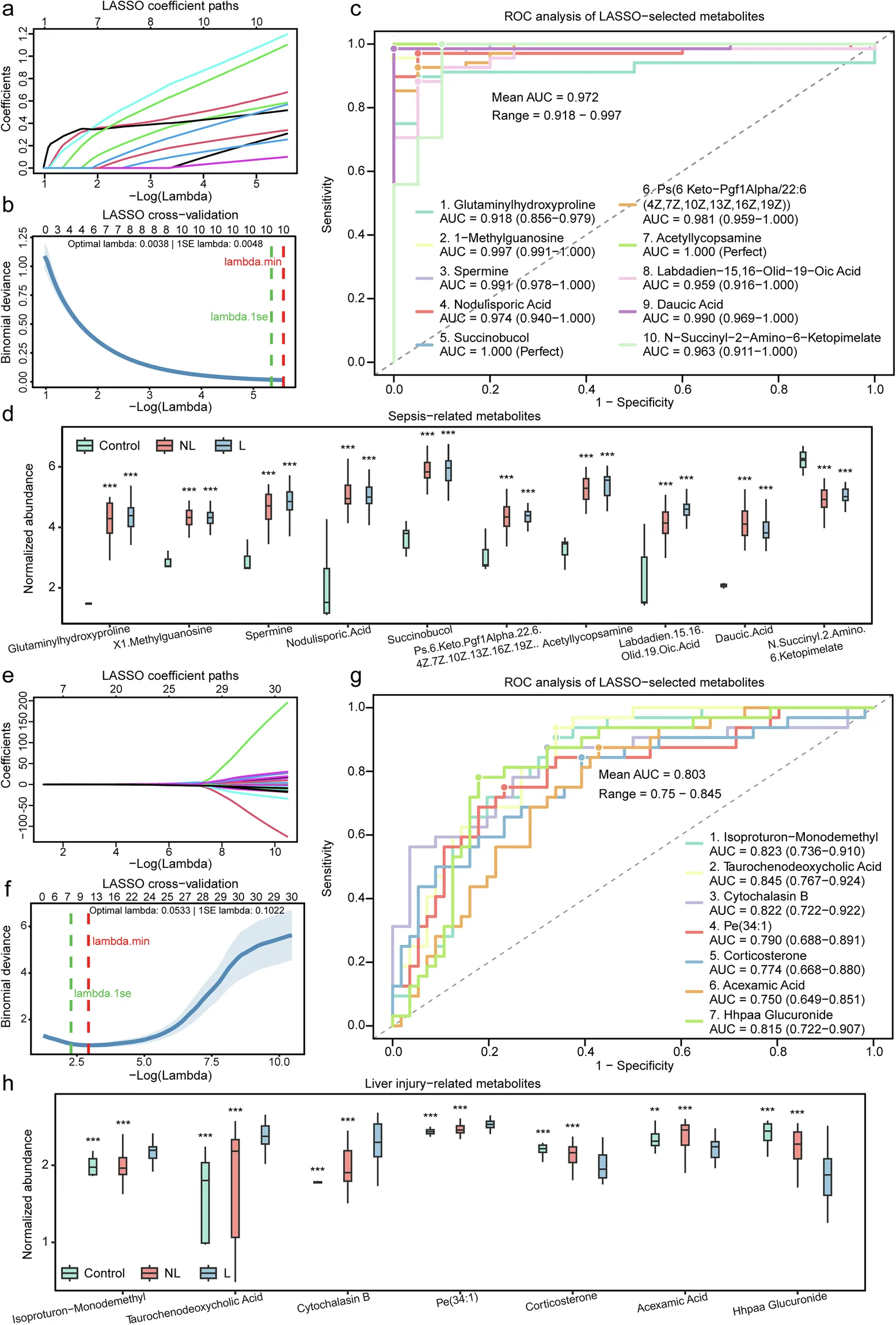
Exploratory classification analysis of LASSO-selected metabolite panels associated with sepsis and liver injury status. (**a, e**) LASSO coefficient profiles of sepsis (**a**) and liver injury (**e**) selected metabolites. (**b, f**) Cross-validation for optimal lambda selection in LASSO regression of sepsis (**b**) and liver injury (**f**) associated metabolites. (**c, g**) The receiver operating characteristic (ROC) curves illustrate exploratory classification analyses of LASSO-selected metabolite panels within the current dataset. (**d, h**) Box plots show the abundance distributions of metabolites included in the exploratory LASSO-derived classification panels for sepsis (**d**) and liver injury status (**h**) across the three clinical groups.

To explore candidate metabolite panels associated with sepsis and liver injury status, Least Absolute Shrinkage and Selection Operator (LASSO) regression analysis was applied to metabolites derived from exploratory analyses. The coefficient profiles revealed distinct variable selection trajectories during regularization for both sepsis-related and liver injury-related metabolites (Fig. 6a, e). Cross-validation was used to determine the optimal lambda values for the sepsis and liver injury models (Fig. 6b, f). The resulting metabolite panels were further evaluated using receiver operating characteristic (ROC) analysis as an exploratory assessment of their discriminatory performance within the current dataset (Fig. 6c, g). These analyses were intended to illustrate potential downstream applications of this dataset rather than to establish validated diagnostic biomarkers. Given the limited sample size and the absence of external validation, the observed discriminatory performance should be interpreted cautiously. Comparative analysis of relative metabolite abundance across groups illustrated abundance patterns among the selected metabolites. The sepsis-associated panel showed different abundance profiles between control and sepsis groups, whereas the liver injury-associated panel showed different abundance profiles among groups with and without liver injury (Fig. 6d, h).

Several metabolites related to bile acid metabolism, nucleotide metabolism, and lipid metabolism exhibited longitudinal abundance changes across clinical groups and time points. These results provide examples of metabolite features and analytical outputs that can be further explored using this dataset^25–28^.

## Methods

### Human ethics statement and blood sample collection

Blood samples from sepsis patients were collected from the Ruijin Hospital. The cohort included 17 sepsis patients, including 9 with liver injury and 8 without liver injury and 20 healthy controls. Eligible sepsis patients were screened and recruited from patients admitted to Ruijin Hospital who fulfilled the study criteria, while healthy controls were enrolled after eligibility confirmation. Longitudinal blood samples were repeatedly collected from the same sepsis patients at four ICU-associated time points (D1, D3, D7, and D14), enabling temporal metabolomic profiling within individuals (Table 7).

**Table 7:** Sample-level metadata of the metabolomics dataset.

Sample_id	Patient_id	Group	Timepoint	Sex	Age	Raw_file_POS	Raw_file_NEG
Control1	Control1	Control	NA	Male	67	POS_Id17YJ_C1.raw	NEG_Id17YJ_C1.raw
Control10	Control10	Control	NA	Male	63	POS_Id17YJ_C10.raw	NEG_Id17YJ_C10.raw
Control11	Control11	Control	NA	Female	62	POS_Id17YJ_C11.raw	NEG_Id17YJ_C11.raw
Control12	Control12	Control	NA	Female	70	POS_Id17YJ_C12.raw	NEG_Id17YJ_C12.raw
Control13	Control13	Control	NA	Female	61	POS_Id17YJ_C13.raw	NEG_Id17YJ_C13.raw
Control14	Control14	Control	NA	Female	64	POS_Id17YJ_C14.raw	NEG_Id17YJ_C14.raw
Control15	Control15	Control	NA	Female	70	POS_Id17YJ_C15.raw	NEG_Id17YJ_C15.raw
Control16	Control16	Control	NA	Female	68	POS_Id17YJ_C16.raw	NEG_Id17YJ_C16.raw
Control17	Control17	Control	NA	Female	70	POS_Id17YJ_C17.raw	NEG_Id17YJ_C17.raw
Control18	Control18	Control	NA	Female	63	POS_Id17YJ_C18.raw	NEG_Id17YJ_C18.raw
Control19	Control19	Control	NA	Female	68	POS_Id17YJ_C19.raw	NEG_Id17YJ_C19.raw
Control2	Control2	Control	NA	Male	63	POS_Id17YJ_C2.raw	NEG_Id17YJ_C2.raw
Control20	Control20	Control	NA	Female	61	POS_Id17YJ_C20.raw	NEG_Id17YJ_C20.raw
Control3	Control3	Control	NA	Male	66	POS_Id17YJ_C3.raw	NEG_Id17YJ_C3.raw
Control4	Control4	Control	NA	Male	67	POS_Id17YJ_C4.raw	NEG_Id17YJ_C4.raw
Control5	Control5	Control	NA	Male	62	POS_Id17YJ_C5.raw	NEG_Id17YJ_C5.raw
Control6	Control6	Control	NA	Male	69	POS_Id17YJ_C6.raw	NEG_Id17YJ_C6.raw
Control7	Control7	Control	NA	Male	65	POS_Id17YJ_C7.raw	NEG_Id17YJ_C7.raw
Control8	Control8	Control	NA	Male	70	POS_Id17YJ_C8.raw	NEG_Id17YJ_C8.raw
Control9	Control9	Control	NA	Male	64	POS_Id17YJ_C9.raw	NEG_Id17YJ_C9.raw
L1_D1	L1	L	D1	Male	56	POS_Id17YJ_L1_D1.raw	NEG_Id17YJ_L1_D1.raw
L1_D14	L1	L	D14	Male	56	POS_Id17YJ_L1_D14.raw	NEG_Id17YJ_L1_D14.raw
L1_D3	L1	L	D3	Male	56	POS_Id17YJ_L1_D3.raw	NEG_Id17YJ_L1_D3.raw
L1_D7	L1	L	D7	Male	56	POS_Id17YJ_L1_D7.raw	NEG_Id17YJ_L1_D7.raw
L2_D1	L2	L	D1	Male	82	POS_Id17YJ_L2_D1.raw	NEG_Id17YJ_L2_D1.raw
L2_D14	L2	L	D14	Male	82	POS_Id17YJ_L2_D14.raw	NEG_Id17YJ_L2_D14.raw
L2_D3	L2	L	D3	Male	82	POS_Id17YJ_L2_D3.raw	NEG_Id17YJ_L2_D3.raw
L2_D7	L2	L	D7	Male	82	POS_Id17YJ_L2_D7.raw	NEG_Id17YJ_L2_D7.raw
L3_D1	L3	L	D1	Male	37	POS_Id17YJ_L3_D1.raw	NEG_Id17YJ_L3_D1.raw
L3_D14	L3	L	D14	Male	37	POS_Id17YJ_L3_D14.raw	NEG_Id17YJ_L3_D14.raw
L3_D3	L3	L	D3	Male	37	POS_Id17YJ_L3_D3.raw	NEG_Id17YJ_L3_D3.raw
L3_D7	L3	L	D7	Male	37	POS_Id17YJ_L3_D7.raw	NEG_Id17YJ_L3_D7.raw
L4_D1	L4	L	D1	Female	76	POS_Id17YJ_L4_D1.raw	NEG_Id17YJ_L4_D1.raw
L4_D14	L4	L	D14	Female	76	POS_Id17YJ_L4_D14.raw	NEG_Id17YJ_L4_D14.raw
L4_D3	L4	L	D3	Female	76	POS_Id17YJ_L4_D3.raw	NEG_Id17YJ_L4_D3.raw
L4_D7	L4	L	D7	Female	76	POS_Id17YJ_L4_D7.raw	NEG_Id17YJ_L4_D7.raw
L5_D1	L5	L	D1	Female	64	POS_Id17YJ_L5_D1.raw	NEG_Id17YJ_L5_D1.raw
L5_D14	L5	L	D14	Female	64	POS_Id17YJ_L5_D14.raw	NEG_Id17YJ_L5_D14.raw
L5_D3	L5	L	D3	Female	64	POS_Id17YJ_L5_D3.raw	NEG_Id17YJ_L5_D3.raw
L5_D7	L5	L	D7	Female	64	POS_Id17YJ_L5_D7.raw	NEG_Id17YJ_L5_D7.raw
L6_D1	L6	L	D1	Female	60	POS_Id17YJ_L6_D1.raw	NEG_Id17YJ_L6_D1.raw
L6_D14	L6	L	D14	Female	60	POS_Id17YJ_L6_D14.raw	NEG_Id17YJ_L6_D14.raw
L6_D3	L6	L	D3	Female	60	POS_Id17YJ_L6_D3.raw	NEG_Id17YJ_L6_D3.raw
L6_D7	L6	L	D7	Female	60	POS_Id17YJ_L6_D7.raw	NEG_Id17YJ_L6_D7.raw
L7_D1	L7	L	D1	Male	51	POS_Id17YJ_L7_D1.raw	NEG_Id17YJ_L7_D1.raw
L7_D14	L7	L	D14	Male	51	POS_Id17YJ_L7_D14.raw	NEG_Id17YJ_L7_D14.raw
L7_D3	L7	L	D3	Male	51	POS_Id17YJ_L7_D3.raw	NEG_Id17YJ_L7_D3.raw
L7_D7	L7	L	D7	Male	51	POS_Id17YJ_L7_D7.raw	NEG_Id17YJ_L7_D7.raw
L8_D1	L8	L	D1	Male	52	POS_Id17YJ_L8_D1.raw	NEG_Id17YJ_L8_D1.raw
L8_D14	L8	L	D14	Male	52	POS_Id17YJ_L8_D14.raw	NEG_Id17YJ_L8_D14.raw
L8_D3	L8	L	D3	Male	52	POS_Id17YJ_L8_D3.raw	NEG_Id17YJ_L8_D3.raw
L8_D7	L8	L	D7	Male	52	POS_Id17YJ_L8_D7.raw	NEG_Id17YJ_L8_D7.raw
NL1_D1	NL1	NL	D1	Female	70	POS_Id17YJ_NL1_D1.raw	NEG_Id17YJ_NL1_D1.raw
NL1_D14	NL1	NL	D14	Female	70	POS_Id17YJ_NL1_D14.raw	NEG_Id17YJ_NL1_D14.raw
NL1_D3	NL1	NL	D3	Female	70	POS_Id17YJ_NL1_D3.raw	NEG_Id17YJ_NL1_D3.raw
NL1_D7	NL1	NL	D7	Female	70	POS_Id17YJ_NL1_D7.raw	NEG_Id17YJ_NL1_D7.raw
NL2_D1	NL2	NL	D1	Male	50	POS_Id17YJ_NL2_D1.raw	NEG_Id17YJ_NL2_D1.raw
NL2_D14	NL2	NL	D14	Male	50	POS_Id17YJ_NL2_D14.raw	NEG_Id17YJ_NL2_D14.raw
NL2_D3	NL2	NL	D3	Male	50	POS_Id17YJ_NL2_D3.raw	NEG_Id17YJ_NL2_D3.raw
NL2_D7	NL2	NL	D7	Male	50	POS_Id17YJ_NL2_D7.raw	NEG_Id17YJ_NL2_D7.raw
NL3_D1	NL3	NL	D1	Male	75	POS_Id17YJ_NL3_D1.raw	NEG_Id17YJ_NL3_D1.raw
NL3_D14	NL3	NL	D14	Male	75	POS_Id17YJ_NL3_D14.raw	NEG_Id17YJ_NL3_D14.raw
NL3_D3	NL3	NL	D3	Male	75	POS_Id17YJ_NL3_D3.raw	NEG_Id17YJ_NL3_D3.raw
NL3_D7	NL3	NL	D7	Male	75	POS_Id17YJ_NL3_D7.raw	NEG_Id17YJ_NL3_D7.raw
NL4_D1	NL4	NL	D1	Female	61	POS_Id17YJ_NL4_D1.raw	NEG_Id17YJ_NL4_D1.raw
NL4_D14	NL4	NL	D14	Female	61	POS_Id17YJ_NL4_D14.raw	NEG_Id17YJ_NL4_D14.raw
NL4_D3	NL4	NL	D3	Female	61	POS_Id17YJ_NL4_D3.raw	NEG_Id17YJ_NL4_D3.raw
NL4_D7	NL4	NL	D7	Female	61	POS_Id17YJ_NL4_D7.raw	NEG_Id17YJ_NL4_D7.raw
NL5_D1	NL5	NL	D1	Male	88	POS_Id17YJ_NL5_D1.raw	NEG_Id17YJ_NL5_D1.raw
NL5_D14	NL5	NL	D14	Male	88	POS_Id17YJ_NL5_D14.raw	NEG_Id17YJ_NL5_D14.raw
NL5_D3	NL5	NL	D3	Male	88	POS_Id17YJ_NL5_D3.raw	NEG_Id17YJ_NL5_D3.raw
NL5_D7	NL5	NL	D7	Male	88	POS_Id17YJ_NL5_D7.raw	NEG_Id17YJ_NL5_D7.raw
NL6_D1	NL6	NL	D1	Male	88	POS_Id17YJ_NL6_D1.raw	NEG_Id17YJ_NL6_D1.raw
NL6_D14	NL6	NL	D14	Male	88	POS_Id17YJ_NL6_D14.raw	NEG_Id17YJ_NL6_D14.raw
NL6_D3	NL6	NL	D3	Male	88	POS_Id17YJ_NL6_D3.raw	NEG_Id17YJ_NL6_D3.raw
NL6_D7	NL6	NL	D7	Male	88	POS_Id17YJ_NL6_D7.raw	NEG_Id17YJ_NL6_D7.raw
NL7_D1	NL7	NL	D1	Male	67	POS_Id17YJ_NL7_D1.raw	NEG_Id17YJ_NL7_D1.raw
NL7_D14	NL7	NL	D14	Male	67	POS_Id17YJ_NL7_D14.raw	NEG_Id17YJ_NL7_D14.raw
NL7_D3	NL7	NL	D3	Male	67	POS_Id17YJ_NL7_D3.raw	NEG_Id17YJ_NL7_D3.raw
NL7_D7	NL7	NL	D7	Male	67	POS_Id17YJ_NL7_D7.raw	NEG_Id17YJ_NL7_D7.raw
NL8_D1	NL8	NL	D1	Female	78	POS_Id17YJ_NL8_D1.raw	NEG_Id17YJ_NL8_D1.raw
NL8_D14	NL8	NL	D14	Female	78	POS_Id17YJ_NL8_D14.raw	NEG_Id17YJ_NL8_D14.raw
NL8_D3	NL8	NL	D3	Female	78	POS_Id17YJ_NL8_D3.raw	NEG_Id17YJ_NL8_D3.raw
NL8_D7	NL8	NL	D7	Female	78	POS_Id17YJ_NL8_D7.raw	NEG_Id17YJ_NL8_D7.raw
NL9_D1	NL9	NL	D1	Female	56	POS_Id17YJ_NL9_D1.raw	NEG_Id17YJ_NL9_D1.raw
NL9_D14	NL9	NL	D14	Female	56	POS_Id17YJ_NL9_D14.raw	NEG_Id17YJ_NL9_D14.raw
NL9_D3	NL9	NL	D3	Female	56	POS_Id17YJ_NL9_D3.raw	NEG_Id17YJ_NL9_D3.raw
NL9_D7	NL9	NL	D7	Female	56	POS_Id17YJ_NL9_D7.raw	NEG_Id17YJ_NL9_D7.raw

All human studies were reviewed and approved by the Ethics Institutional Review Board of Ruijin Hospital, Shanghai Jiao Tong University School of Medicine, with clinical ethical approval number K2025-91. The study was conducted in accordance with the principles of the Declaration of Helsinki and under the supervision of the institutional ethics committee. Before any study-related sample collection, written informed consent was obtained from all participants or, when participants were unable to provide consent because of critical illness, from their legally authorized representatives. Trained study investigators explained the study purpose, procedures, potential risks and benefits, voluntary nature of participation, and the right to withdraw from the study at any time without affecting clinical care.

Participants consented to participation in the study, collection and use of blood samples, extraction of relevant clinical information, LC-MS-based metabolomic analysis, publication of aggregated research findings, and sharing of de-identified metabolomics data and associated sample-level metadata for scientific reuse. To protect participant confidentiality, all samples and clinical metadata were assigned study-specific coded identifiers. Direct personal identifiers were removed before data analysis and before deposition in public repositories. The key linking coded identifiers to personal information was stored securely and was accessible only to authorized study personnel. Clinical records and consent documents were stored in secured institutional locations, and electronic data were maintained on password-protected systems. Only de-identified data were used for analysis, reporting, and public data sharing.

Sepsis was determined using Sepsis-3 criteria, with organ dysfunction defined as a SOFA score of 2 or more points. To account for the pathophysiologic alterations in sepsis, we applied Modified Drug-Induced Liver Injury (DILI) Criteria to identify sepsis patients with liver injury within the first day after sepsis diagnosis (i.e., upon the first fulfilment of Sepsis-3 criteria). sepsis-associated liver injury was considered present if at least one of the following conditions was met: an ALT level of at least five times the upper limit of normal (ULN); an ALP level of at least twice the ULN; a combination of serum total bilirubin of at least 2.0 mg/dL and ALT at least three times the ULN; or a combination of serum total bilirubin of at least 2.0 mg/dL and ALP at or above the ULN.

### Untargeted metabolomic sample preparation

100 μL liquid sample was added to a 1.5 mL centrifuge tube with 800 μL solution (acetonitrile: methanol = 1:1(v: v)) containing four internal standards (0.02 mg/mL L-2-chlorophenylalanine, etc.) to extract metabolites. The samples were mixed by vortex for 30 s and low-temperature sonicated for 30 min (5 °C, 40 KHz). The samples were placed at −20 °C for 30 min to precipitate the proteins. Then the samples were centrifuged for 15 min (4 °C, 13000 g). The supernatant was removed and blown dry under nitrogen. The sample was then re-solubilized with 100 µL solution (acetonitrile: water = 1:1) and extracted by low-temperature ultrasonication for 5 min (5 °C, 40 KHz), followed by centrifugation at 13000 g and 4 °C for 10 min. The supernatant was transferred to sample vials for LC-MS/MS analysis.

### UHPLC-MS/MS analysis

The LC-MS/MS analysis of sample was conducted on a UHPLC-Q Exactive system equipped with an ACQUITY HSS T3 column (100 mm × 2.1 mm i.d., 1.8 μm; Waters, USA) at Majorbio Bio-Pharm Technology Co. Ltd. (Shanghai, China). The mobile phases consisted of 0.1% formic acid in water: acetonitrile (2:98, v/v) (solvent A) and 0.1% formic acid in acetonitrile (solvent B). The flow rate was 0.40 mL/min and the column temperature was 40 °C. The injection volume was 5 μL.

MS conditions: the UHPLC system was coupled to a UHPLC-Q Exactive system Mass Spectrometer equipped with an electrospray ionization (ESI) source operating in positive mode and negative mode. The optimal conditions were set as followsed: source temperature, 400 °C; sheath gas flow rate 40 arbitrary units (arb); aux gas flow rate, 10 arb; and ion-spray voltage floating (ISVF),-2800V in negative-ion mode. The detection was carried out over a mass range of 70–1050 m/z.

### Unsupervised exploratory analysis: principal component analysis

PCA was performed as an unsupervised exploratory analysis to visualize global metabolic variation and evaluate overall sample clustering patterns without prior group assignment. PCA was conducted using the factoextra package (v1.0.7)^29^ based on metabolite abundance matrices. Inter-sample reproducibility was further assessed by calculating Spearman’s correlation coefficients and constructing correlation heatmaps.

Because PCA and correlation analyses were used primarily for data exploration and quality assessment, no formal hypothesis testing or statistical significance evaluation was performed at this stage.

### Supervised exploratory discriminant analysis: orthogonal partial least squares-discriminant analysis

Inter-group metabolic differences were explored using OPLS-DA implemented in the ropls package (v1.38.0)^30^. This supervised exploratory analysis was used to identify metabolites contributing to metabolic separation among groups.

Differential metabolites were screened using the criteria of variable importance in projection (VIP) > 1 and unadjusted *P*-values < 0.05 derived from Student’s t-tests. Volcano plots were generated using the scRNAtoolVis package (v0.0.7)^31^ to visualize metabolite distributions across comparison groups.

Because these analyses were intended for exploratory metabolite discovery and dataset characterization, no multiple-testing correction was applied.

### Exploratory temporal pattern analysis: fuzzy c-means clustering

To characterize longitudinal metabolic dynamics, fuzzy c-means clustering analysis was performed using the Mfuzz package (v2.66.0)^32^. Group-averaged metabolite abundance values were normalized using Z-score transformation prior to clustering. Metabolites were subsequently classified into four temporal expression clusters according to their longitudinal abundance trajectories across D1, D3, D7, and D14 time points.

As this analysis was intended for exploratory temporal pattern characterization and biological hypothesis generation, no multiple-testing correction was applied.

### Exploratory network analysis: weighted correlation network analysis

WGCNA was conducted using the WGCNA package (v1.73)^33^ to identify co-abundance metabolite modules associated with clinical phenotypes. The input matrix consisted of 2,894 annotated metabolites derived from LC-MS metabolomics profiling.

Soft-threshold powers ranging from 1 to 20 were evaluated to determine the optimal network construction parameter. The correlation matrix was transformed into an adjacency matrix and subsequently into a topological overlap matrix (TOM). Metabolite modules were identified using average linkage hierarchical clustering with a minimum module size of 30 metabolites, followed by merging of highly similar modules.

Associations between module eigengenes and clinical phenotypes were evaluated using Pearson correlation analysis, with corresponding *P*-values calculated based on Student’s t-distribution. Raw, unadjusted *P*-values < 0.05 were used to identify phenotype-associated modules. Module membership (MM) was defined as the Pearson correlation coefficient between individual metabolite abundance profiles and corresponding module eigengenes.

Because WGCNA was used as an exploratory network-based feature discovery approach, multiple-testing correction was not applied.

### Pathway enrichment analysis

Pathway enrichment analyses were conducted using OmicShare tools^34^ based on the Kyoto Encyclopedia of Genes and Genomes (KEGG) database. Enrichment analyses were applied to differential metabolites identified from OPLS-DA, temporal metabolite clusters derived from Mfuzz analysis, and intersected metabolite signatures identified through integrative analyses.

Significantly enriched pathways were identified using Fisher’s exact tests with raw, unadjusted *P*-values < 0.05. Enrichment results were visualized using bubble plots and bidirectional bar charts.

Because these analyses were intended for exploratory biological interpretation and pathway characterization, multiple-testing correction was not applied.

### Integrative exploratory metabolite signature identification

To identify exploratory metabolic signatures associated with sepsis and sepsis-associated liver injury, metabolites identified from differential metabolite analysis, temporal clustering, and WGCNA were integrated through overlap analyses. Positively and negatively associated metabolite signatures were defined based on consistent trends across multiple analytical approaches.

These integrated metabolite signatures were subsequently used for downstream feature prioritization and diagnostic modeling.

### Regularized regression modeling and feature prioritization

Least Absolute Shrinkage and Selection Operator (LASSO) regression analysis was performed using the glmnet package (v4.1-10)^35^ to prioritize candidate metabolite features for exploratory classification analyses of sepsis and sepsis-associated liver injury.

Metabolites previously identified through exploratory analyses were first Z-score normalized. Negatively associated metabolites were sign-inverted prior to integration with positively associated metabolites to preserve biological directionality during model construction.

A 10-fold cross-validated LASSO logistic regression model was implemented using deviance minimization, with disease status used as the response variable and metabolite abundance profiles used as predictors. Optimal regularization parameters (lambda.min and lambda.1se) were selected based on cross-validation curves, and metabolites with non-zero coefficients at the selected lambda values were retained.

Given the relatively limited sample size and absence of an external validation cohort, these analyses should be interpreted as exploratory feature prioritization rather than definitive clinical biomarker validation.

### Exploratory classification performance visualization and statistical assessment

Receiver operating characteristic (ROC) analyses were performed using the pROC package (v1.19.0.1)^36^ to visualize the classification performance of selected metabolite panels within the current dataset by calculating area under the curve (AUC) values and corresponding 95% confidence intervals.

Relative metabolite abundance differences among healthy controls, sepsis patients without liver injury, and sepsis patients with liver injury were visualized using ggplot2 (v3.5.2)^37^ boxplots. Pairwise group comparisons were assessed using Wilcoxon rank-sum tests with raw, unadjusted *P*-values.

These analyses were intended primarily for descriptive visualization and preliminary evaluation of metabolite distribution patterns within the current dataset.

## Data Records

All metabolomics raw data and associated processed files generated in this study have been deposited in the MetaboLights^38^ repository under accession number MTBLS14459^39^ (https://www.ebi.ac.uk/metabolights/MTBLS14459). The deposited dataset includes raw LC-MS files, processed metabolite abundance matrices, metabolite annotation tables, quality control metrics, and pathway annotation resources to facilitate data transparency and reuse.

The repository is organized into two primary directories: FILES/RAW_FILES and FILES/SUPPLEMENTARY_FILES.

FILES/RAW_FILES contains the original LC-MS raw data generated from both positive and negative ionization modes (.raw format). Specifically, the dataset includes 95 positive-ion raw files and 95 negative-ion raw files corresponding to 20 healthy controls, 8 sepsis patients without liver injury sampled at four longitudinal time points (32 samples), 9 sepsis patients with liver injury sampled at four longitudinal time points (36 samples), and 7 pooled quality control (QC) samples. Raw file names correspond directly to the sample identifiers provided in Table 7, thereby enabling explicit linkage between raw data files and associated metadata.

FILES/SUPPLEMENTARY_FILES contains eight supplementary data files:

(1) POS_md5sum.txt and (2) NEG_md5sum.txt

These files provide MD5 checksum values for all positive- and negative-ion raw LC-MS files to ensure file integrity verification after download.

(3) KEGG_background_metabolite_file.xlsx

This file contains the metabolite background dataset used for KEGG pathway enrichment analysis, including metabolite identifiers, KEGG Compound IDs, and associated pathway annotations.

(4) 2894_metab_abund.csv

This file contains the normalized abundance matrix of the 2,894 annotated metabolites retained after preprocessing and quality filtering. Rows correspond to metabolites and columns correspond to biological and QC samples. Each cell contains the normalized ion intensity value for the corresponding metabolite-sample pair.

(5) all2894metab_abund.csv

This comprehensive annotation table contains the abundance information for all 2,894 annotated metabolites together with detailed metabolite annotation metadata. Included variables comprise metab_id, Metabolite, Compound ID, m/z, Retention time, ionization Mode, Adducts, Formula, Fragmentation Score, Theoretical Fragmentation Score, annotation confidence level, Mass Error (ppm), KEGG Compound ID, Library ID, compound_source, CAS ID, DrugBank identifiers, structural SMILES information, and normalized abundance values for all 95 samples.

(6) all_metab_data.txt

This file contains the complete processed feature table for all 18,247 high-quality metabolic features retained after preprocessing. In addition to metabolite abundance values across all 95 samples, the table includes feature annotation information such as metab_id, m/z, retention time, ionization mode, adduct type, annotation confidence level, spectral matching scores, and database identifiers.

(7) metab_abund.txt

This file contains the abundance matrix for all 18,247 processed metabolic features. Rows correspond to metabolic features and columns correspond to all biological and QC samples.

(8) metab_abund.txt_rsd.txt

This file contains relative standard deviation (RSD) values calculated from pooled QC samples for all detected metabolic features. The first column represents metab_id and the second column contains the corresponding RSD value, enabling users to independently assess analytical reproducibility and feature stability.

Essential sample metadata, preprocessing information, and metabolite annotation summaries are provided in Tables 8–9.

**Table 8:** Data preprocessing and quality filtering summary.

Ion mode	pos	neg	mix
**Effective peak (raw)**	16180	17359	33539
**Identified metabolites (raw)**	2067	2363	4430
**Percentage**	12.78%	13.61%	13.21%
**Effective peak (origin)**	8329	9918	18247
**Identified metabolites (origin)**	1339	1555	2894
**Percentage**	16.08%	15.68%	15.86%

‘Raw’ refers to the original table, whilst ‘Origin’ refers to the table resulting from data pre-processing of the raw table (see the Technical Validation section for details of the pre-processing methods).

**Table 9:** Metabolite annotation information.

Database	Number	Percentage of the total number of metabolites identified
Metabolites annotated in KEGG (marked with a ‘C’)	935	32.31%
Metabolites annotated in HMDB (marked with an ‘H’)	2210	76.36%
Metabolites annotated in KEGG pathways (marked with a ‘map’)	456	15.76%

## Technical Validation

### Data Preprocessing and Feature Detection

Raw mass spectrometry data were systematically processed through complementary qualitative and quantitative workflows, with positive and negative ionization modes analyzed independently using identical parameters. Initially, 33,539 valid ion features were detected across both ionization modes, corresponding to 4,430 annotated metabolites. Following data preprocessing, quality filtering, normalization, and removal of low-quality variables, 18,247 high-quality peaks with distinct chromatographic profiles and uniform signal distribution were retained for downstream analyses.

### Metabolite Annotation

For qualitative metabolite annotation, raw data were first converted using ProteoWizard and subsequently analyzed using Progenesis QI (Waters Corporation, Milford, USA) for peak alignment, database searching, and metabolite annotation. MS and MS/MS spectra were matched against multiple metabolite databases, including the Human Metabolome Database (HMDB), Metlin, KEGG, and a proprietary in-house spectral library.

Database matching identified 2,894 metabolites for further investigation. Annotation confidence was classified into two MSI-compatible categories: 1,411 metabolites were assigned as high-confidence annotations [B(i)], based on accurate matching against experimentally acquired MS/MS spectra from the in-house spectral library; the remaining 1,483 metabolites were classified as putative annotations [B(ii)], based on matching against computationally predicted or publicly available MS/MS spectral libraries, including theoretical databases (Tables 8–9). To further improve metabolite interpretability and facilitate data reuse, hierarchical classification and pathway annotation were systematically summarized based on HMDB, KEGG Compound, and KEGG PATHWAY databases.

### Quality Control Procedures

Quality control (QC) procedures were implemented throughout sample preparation, LC-MS/MS acquisition, and data preprocessing to monitor analytical stability and ensure reproducibility. A pooled QC sample was prepared by mixing equal aliquots from all study samples to represent the overall metabolite composition of the dataset. QC samples were processed identically to biological samples and injected at regular intervals (every 10 analytical samples) throughout the acquisition sequence to continuously monitor instrument stability and signal reproducibility.

### Quantitative Data Processing and Normalization

Quantitative data processing was performed using Progenesis QI and included baseline correction, peak picking, peak integration, retention time alignment, and peak alignment to generate a comprehensive feature matrix containing retention time, mass-to-charge ratio (m/z), and peak intensity information. Features with excessive missing values were filtered prior to analysis; remaining missing values were imputed using minimum detectable values. Total ion sum scaling normalization was subsequently applied to reduce systematic variations associated with sample preparation and instrumental fluctuations. Finally, log₁₀ transformation was applied to reduce heteroscedasticity and improve approximation to a normal distribution for downstream statistical analyses.

### Analytical Reproducibility Assessment

Analytical reproducibility was assessed using pooled QC samples by calculating the relative standard deviation (RSD) of each detected feature. Variables exhibiting RSD values greater than 30% across QC samples were excluded from downstream analyses. After quality filtering, 18,247 high-quality metabolic features with stable chromatographic behavior and uniform signal distribution were retained for subsequent analyses. No obvious batch effects or analytical signal drift were observed during LC-MS/MS acquisition based on QC sample distributions and chromatographic consistency assessments. The overall quality and stability of the dataset were further supported by tight clustering of QC samples in PCA space and high inter-sample correlations observed among biological replicates (Fig. 2a–b).

## Usage Notes

This dataset provides a longitudinal metabolomics resource for characterizing temporal metabolite abundance patterns during sepsis and sepsis-associated liver injury. The inclusion of serial blood samples collected at multiple ICU time points, together with comprehensive clinical metadata and quality-controlled LC-MS profiling, enables diverse downstream applications.

Potential reuse scenarios include:

1. longitudinal trajectory analysis of metabolic alterations during sepsis progression;
2. evaluation of candidate metabolites associated with liver injury;
3. integrative analyses combining metabolomics data with transcriptomics, proteomics, or clinical phenotypes;
4. systems-level analyses of metabolomics data in critical illness;
5. development and benchmarking of computational methods for metabolomics data analysis.

In addition to raw LC-MS files, the repository includes processed metabolite abundance matrices, metabolite annotations, clinical metadata, and bioinformatics analysis scripts to facilitate reproducibility and data reuse.

Because of the relatively small cohort size and repeated sampling structure, the longitudinal analyses in this study should primarily be interpreted as exploratory and descriptive. Although temporal clustering and group-level comparisons were performed to characterize metabolic variation across ICU-associated time points, formal repeated-measures longitudinal modeling approaches (e.g., mixed-effects models or generalized estimating equations) were not implemented. In particular, the LASSO-derived metabolite panels and corresponding ROC performance metrics were generated within the same dataset and were not externally validated, which may increase the risk of overfitting. Therefore, the dataset is particularly suitable for future methodological development, longitudinal validation studies, and integrative analyses using larger independent cohorts. Accordingly, the analytical results presented in this Data Descriptor should primarily be regarded as illustrative examples demonstrating potential uses of the dataset, rather than definitive biological or clinical conclusions.

## Supplementary information

Supplementary Tables

## Data Availability

Code files are available from the GitHub repository^40^ https://github.com/Dong-Leng/SepsisLiMetDB. All the bioinformatics analyses were performed in R 4.4.2 on Windows 11 (64-bit) platform. The following software packages were used for the analyses: factoextra v1.0.7, ropls v1.38.0, scRNAtoolVis v0.0.7, ggpubr v0.6.1, ggthemes v5.1.0, ggplot2 v3.5.2, ggforce v0.5.0, Mfuzz v2.66.0, dplyr v1.1.4, tidyr v1.3.1, pheatmap v1.0.13, glmnet v4.1-10, pROC v1.19.0.1, reshape2 v1.4.4, WGCNA v1.73, ggsignif v0.6.4, ggbeeswarm v0.7.2, stringr v1.5.1, UpSetR v1.4.0, RColorBrewer v1.1-3.
